# Royal College of Surgeons Council Visit to Bristol

**Published:** 1991-03

**Authors:** A. J. Webb

**Affiliations:** Consultant Surgeon, Bristol Royal Infirmary in the Great Hall, University of Bristol on Friday 9th November 1990


### Royal College of Surgeons Council Visit to Bristol

Highlights from the after dinner address, proposing the College Toast delivered by A.J. Webb, Consultant Surgeon, Bristol Royal Infirmary in the Great Hall, University of Bristol on Friday 9th November 1990.

Sir John, Lady Kingman, Mr. President, Members of Council, Honoured Guests and Fellows of the College.

Mr. President, having with a goodly number of your Council graced us with your presence today, it would be improper for me not to say something of our City and this particular location. The mayoral role dates from 1261, just one year later than London. Our most important charter dates from 1373 a gift from Edward III in recognition of support at the Siege of Calais.

Pre-Roman Celtic settlements are residual on Clifton Down and Leigh Woods – very close to the St. Vincent Rocks Hotel where I gather some of the Council are staying. During Roman times there is a connection also. The Fosseway joined Lincoln to Leicester and Cirencester (Corinium) to Bath (Aquae Sulis) then to Exeter. There existed an extension from Bath to a small port on the River Avon where it is joined by the River Trym, named Abona. A small Roman Town. It is now part of a suburb of Bristol – Sea Mills. It supported the 2nd Augustan Legion in its attempted conquest of Wales (60–70 AD).

The English Chronicle details a battle in 577 AD between Saxons and local Celts at Deorham near Bath. If you drive here from London on the M4, just beyond the crest of the A46 junction, it lies over on your left. The Chronicle fails to mention Bristol in any name form. A silver coin preserved in the Royal Swedish collection at Stockholm bears the image of 'Ethelred Unrede' (Ethelred II nd) printed by Aelfwerd of Brigstowe or Bridge Place later transposed to Bristol. The coin is dated 978 AD so Bristol was founded between 577 and 978 AD.

Close to where we are sitting was one the Parliamentarian Forts for local defence. On 25th July 1643, Col. Henry Washington, a very distant ancestor of George, broke through with his Royalists and dashed down Park Street, as we now know it, forcing Col. Fiennes, then Roundhead Governor of Bristol to surrender the city.

In 1645, September 10th, Col. Fairfax accompanied by Cromwell recaptured Bristol. We have our Cromwell Road, Fairfax and Rupert Street. The Norman Castle was destroyed in 1655 but a small remnant is being excavated on the direct walking route from the BRI to the Avon Breast Screening Unit at Tower Hill.

Two famous diarists visited Bristol. The rather staid John Evelyn came in 1654 and was impressed with Bristol's trade and the profusion of sugar refineries. He compared us with London for the manner of buildings, shops, bridges and the exchange. The naughty Sam' Pepys came over from Bath on 13.7.1668 travelling with Mrs. Pepys and a pretty maid, Deb. Deb Willet was a Bristol girl and later the object of his amorous adventures. His diary reads in paraphrase:-

"Went to see the Quay – a large and noble place – shops were being built there. Deb came back with her Uncle Butts, a sober merchant, very good company and so like one of our sober wealthy London merchants as pleased me mightily. And so brought us back by surprise to his house and did give a good entertainment of strawberries, a whole venison pasty and plenty of brave wine and above all Bristol Milk (sherry)". He goes on – "Walked with my wife and people through the City which is in every respect another London, that one can hardly know it stands in the country".

In the 18th Century, Bristol overtook Norwich to become the largest and richest city next to London and then its position fell away. But it has always been a haven. We have Welsh, Scots, Irish, North and South, and refugees from Poland, Italy, Spain, Hungary, the West Indies, London especially Lloyd's Bank and many others. Bristol has never been over parochial especially in medical matters. Consequently there are very few Bristolian trained or true Bristolians on the hospital staff. This broad approach renders it a vital and lively professional environment.

Mr. President, although on the 25th June 1940, air raids began on Bristol and the devastating daylight raid on Filton was on 25th September; concentrated night bombing, or the 'Blitz', commenced 50 years ago this month on Sunday 24th November. It is not widely realised how severely Bristol suffered. It sustained the most concentrated bombing of any British city, but these facts were censored and obscured at the time. There were 563 air raid alerts – 1,300 people killed – 3,300 wounded – 3,000 houses destroyed and 90,000 houses damaged. On that Sunday the centre of the old city was destroyed, largely by incendiaries. This Great Hall was all but flattened, the beautiful wooden hammer beam roof and panelling with many books lodged here from King's College London were burned: a dreadful loss.

One of the worse raids took place on Good Friday 11th April 1941 – a pleasant sunny day as I recall. The raid began at 8 p.m. and ended at 5.00 a.m. in the morning. The day after, Winston Churchill, our Chancellor, with his entourage, came here for a Degree Congregation. The University Staff, who had mostly been up all night, were alerted by phone to assemble. The University and around had been heavily bombed. They robed in the Committee Room, picked their way in procession over the hoses to the Reception Room where Winston conferred Honorary Doctorates of Law on John G. Winant, the American Ambassador and Robert Menzies, Premier of Australia. There is a photograph of this group standing outside the Wills Building.

As Professor MacInnes wrote 'Seldom in the history of Universities has a ceremony taken place amid surroundings so unusual'. That morning I, like so many other school boys was out and around on my bike. I saw Churchill arrive here.

Later, the party toured Bedminster which had suffered badly in all the 'Blitzes' and Churchill with the others were booed. These were dark days. A journalist gave him a card and pen, requesting one sentence for Bristol. He wrote:- "Carry on all will be well", and it was. Later, on 21.4.45, he returned in triumph. He was invested with the Freedom of the City and at a Degree Congregation conferred Doctorates on A.V. Alexander and Ernie Bevin (both Bristolians). Happily there are many photographs of Bristol's history from the mid 1899's. It has been written that, no printed records of comparable value exists for any other English city.

Mr. President, we are all aware of the heavy burdens on your shoulders and those of your Council. We are living in disturbing times and 1990 has not been a good year so far. 'Thought for the Day' is one of my favourite radio programmes. The Bishop of Oxford, Richard Harris, said recently "Crime is up, armed crime is up, we seem to be following America". Sadly we seem to be following America in the pattern of medical litigation also and unless something drastic is done the prospect is awful. Last week's 'Hospital Doctor' – my regular Friday lunchtime reading, reported two young house officers up on criminal charges for the death of a terminal patient. No other profession suffers the risk of Quadruple Indemnity. Walking to my office last week one of our male staff nurses came rushing past, carrying amongst other things his bicycle wheel and saddle. Some Consultant Pathologists take their bicycles to their offices. Hospital theft is endemic and increasing. Pressures from disorganised wards, lack of consistent nursing staff and limited operating time and so many changes, are demoralising.

I would ask that in your deliberations, you and your colleagues bear in mind those pressures on all surgeons but particularly consultants. I hope that Almighty God will continue to endow you with a good portion of wisdom to enable you to conduct the affairs of surgery aright.

On behalf of the Bristol Surgical Fellows at all levels I thank you and your Council very much for visiting us and ensuring such a rewarding and unforgettable day.

Ladies and Gentlemen please rise and support the Toast which is:-

The Royal College of Surgeons of England coupled with the name of the President, Terence English* together with his Council members here present.

*Mr. Terence English was subsequently Knighted in the 1991 New Year Honours List.

**REFERENCES**

BRISTOL BLITZ DIARY (1982) John Dike, Redcliffe, Bristol.
BRISTOL IN THE FIFTIES (1988) as remembered by James Belsey et al., Redcliffe, Bristol.
BRISTOL – ENGLAND (1950) Harold G. Brown. Brothers, Bristol.
BRISTOL AS IT WAS 1940–1960 (1988). Reece Winston House, Churchill, Bristol.
BRISTOL AT WAR (1962) C.M. MacInnes. London Museum Press Limited.

